# Reversing Neuromuscular Blockade without Nerve Stimulator Guidance in a Postsurgical ICU—An Observational Study

**DOI:** 10.3390/jcm12093253

**Published:** 2023-05-02

**Authors:** Andrea Calef, Rashel Castelgrande, Kristin Crawley, Sara Dorris, Joanna Durham, Kaitlin Lee, Jen Paras, Kristen Piazza, Abigail Race, Laura Rider, Michael Shelley, Emily Stewart, Miranda Tamok, Jennifer Tate, Jeffrey M. Dodd-o

**Affiliations:** 1 Department of Surgery, Johns Hopkins Hospital, Baltimore, MD 21287, USA; 2 Department of Surgery, Anne Arundel Medical Center, Anne Arundel, MD 21401, USA; 3 Department of Surgery, Medstar Medical Group, Baltimore, MD 21201, USA; 4 Department of Surgery, INOVA Fairfax Hospital, Fairfax, VA 22042, USA; 5 Department of Surgery, University of Maryland St Joseph Hospital, Baltimore, MD 21201, USA; 6 Department of Surgery, North Shore University Hospital, Manhasset, NY 11030, USA; 7 Department of Surgery, Maine Medical Center, Portland, ME 04103, USA; 8 Department of Anesthesiology, Johns Hopkins Hospital, Baltimore, MD 21287, USA

**Keywords:** Neuromuscular blockade reversals, neostigmine, residual neuromuscular blockade

## Abstract

We aimed to determine if not using residual neuromuscular blockade (RNB) analysis to guide neuromuscular blockade reversal administration in the postsurgical ICU resulted in consequences related to residual weakness. This single-center, prospective study evaluated 104 patients arriving in a postcardiac surgical ICU. After demonstrating spontaneous movement and T > 35.5 °C, all patients underwent RNB evaluation, and neostigmine/glycopyrrolate was then administered. When patients later demonstrated an adequate Rapid Shallow Breathing Index, negative inspiratory force generation, and arterial blood gas values with minimal mechanical ventilatory support, RNB evaluation was repeated in 94 of the 104 patients, and all patients were extubated. Though RNB evaluation was performed, patients were extubated without considering these results. Eleven of one hundred four patients had not achieved a Train-of-Four (TOF) count of four prior to receiving neostigmine. Twenty of ninety-four patients demonstrated a TOF ratio ≤ 90% prior to extubation. Three patients received unplanned postextubation adjunct respiratory support—one for obvious respiratory weakness, one for pain-related splinting compounding baseline disordered breathing but without obvious benefit from BiPAP, and one for a new issue requiring surgery. Residual neuromuscular weakness may have been unrecognized before extubation in 1 of 104 patients administered neostigmine without RNB analysis. ICU-level care may mitigate consequences in such cases.

## 1. Introduction 

Standard anesthesiology teaching is to utilize residual neuromuscular blockade (RNB) analysis at two points to guide the appropriate administration of agents to reverse neuromuscular blockades given intraoperatively. First, utilize it before administering reversal agents to assure adequate spontaneous resolution of the blockade. Second, utilize it after administering reversal agents to assure the blockade is sufficiently resolved before extubating. Though this is not universally the standard practice before extubating patients in the intensive care unit (ICU), patient safety implications of postoperative residual weakness following neuromuscular blocking agent administration [1] are leading many to urge broader incorporation of such standards [2]. This is based on observed consequences associated with a lack of verifying chemical neuromuscular agent reversal prior to extubation in the operating room [3]. 

The purpose of this study was to evaluate the effectiveness at preventing weakness-induced complications of utilizing neostigmine/glycopyrrolate without RNB analysis to reverse chemical neuromuscular blockades in a surgical ICU. 

## 2. Methods

### 2.1. Study Participants

The study was a prospective, single-center, observational study conducted at an academic hospital 18-bed cardiac surgical ICU. The study was designed to incorporate 100 patients over two years as part of a quality improvement initiative, but the enrollment period was extended due to COVID-19. Patients were eligible August 2017–February 2021 if they were admitted intubated to the ICU directly following sternotomy and heart surgery, insertion of mechanical ventricular support, or heart transplantation, and had received rocuronium and/or vecuronium and had not yet received chemical neuromuscular blocking reversal agents. The ICU is administratively run by the Cardiac Surgical Department but staffed by intensivists from the Departments of Cardiac Surgery, Anesthesiology and Pulmonary Medicine. By practice, some intensivists prefer acetylcholinesterase inhibitors nearly exclusively, while others prefer cyclodextrins to reverse chemical neuromuscular blocking agents. Only patients receiving acetylcholinesterase inhibitors were eligible for this study. 

### 2.2. Baseline Characteristics and Follow-Up

All data except TOF information were extracted from the medical records. Difficult laryngoscopy was defined by Cormack–Lehane Classification [4] >3 by direct laryngoscopy or requirement for adjuvant instrumentation (e.g., bougie, indirect laryngoscopy). Renal dysfunction was considered as baseline creatinine > 2.0. Patients at risk for hepatic dysfunction were considered those with a history of significant ETOH or illicit drug use, known hepatitis of nonalcoholic fatty liver, chronic highly active antiretroviral therapy (HAART), or increased likelihood of embolic disease from endocarditis. Postextubation respiratory adjuncts were considered to be a use of simple nasal cannula oxygen > 6 L/min, simple face mask oxygen > 6 L/min, high-flow nasal cannula, or noninvasive or invasive mechanical support. Such adjuvant therapy was deemed to not be treating muscular weakness if it was administered (1) as HFNC in order to supply epoprostenol for RV support; (2) as HFNC for hypoxia in the setting of pH ≥ 7.35; (3) for a diagnosis of hypervolemia; (4) as planned continuation of patient’s home OSA therapy; (5) in a patient who is able to stand. RNB data were recorded separately as part of a quality improvement initiative. 

### 2.3. Intraoperative and Perioperative Anesthesia

An opioid-sparing anesthetic plan was employed intraoperatively, as previously [5,6]. In short, this involved preinduction administration of acetaminophen (1000 mg) and gabapentin (300–600 mg), intraoperative administration of ketamine (0.2–0.3 mg/kg/h) and/or dexmetetomidine (0.2–1.5 mcg/kg/h, titrated to hemodynamic and sedation goals), and rare use of regional nerve block (Serratus Anterior Plane block). ICU sedation was based predominantly on dexmetetomidine (0.2–1.5 mcg/kg/h) and/or propofol (10–50 mcg/kg/min). Narcotic supplementation per provider choice included fentanyl (≤250 mcg) or hydromophone (≤2 mg) intraoperatively and fentanyl (≤200 mcg) or hydromorphone (≤1 mg) postoperatively prior to extubation. The postoperative sedation target was typically to maintain hemodynamics but minimize spontaneous movement until chest tube bleeding < 150 mL/h was achieved. At this point, sedation was lightened to achieve calm response to commands. After neostigmine administration (see below), sedation was severely limited or completely stopped in an effort to promote awakening and evaluation of extubation readiness. 

Intraoperative muscle relaxant was administered per provider discretion but rarely guided by quantitative RNB evaluation. Intraoperative temperature control typically involves “drifting” and active cooling rarely to a low of ≥33 °C during cardiopulmonary bypass. After cardiopulmonary bypass, patients were actively warmed to 35 °C (bladder temperature) before separating cardiopulmonary bypass. They continued active warming to 36.5 °C in the operating room and in the ICU and did not receive neostigmine/glycopyrrolate until a bladder temperature of 36 °C was achieved.

### 2.4. Neostigmine Administration

Midlevel providers (nurse practitioners and physician assistants) dosed neuromuscular reversal agents when hemodynamic lability and bleeding were resolved (<150 mL/h), the patient has achieved T ≥ 35.5 °C, and has demonstrated some spontaneous movement (eg. extremity movement, respiratory effort). The typical reversal dose is 0.05–0.07 mg/kg neostigmine and 0.01–0.015 mg/kg glycopyrrolate. 

### 2.5. Residual Neuromuscular Blockade Assessment

RNB was objectively assessed using STIMPOD NMS450 acceleromyograph, with leads over the ulnar nerve at the wrist and an accelerometer on the thumb, with the observer placing three of his/her fingers between the thumb and index finger of the patient to apply a mild “stretch”. A Train-of-Four (TOF) stimulation with 60 mV at a 2 Hz frequency was delivered, with each twitch corresponding to a bar on the monitor display. For those achieving a Train-of-Four Count (TOFC) of 4, relative acceleration of 4th vs. 1st twitch (displayed in percentage) was indicated and used as Train-of-Four ratio (TOFR). Assessment was at two time points—once immediately before the delivery of chemical reversal agents for neuromuscular blockers and once before extubation. The second assessment ideally occurred as close to the point of extubation as comfortably possible for the patient (i.e., before propofol, dexmetetomidine, fentanyl, and/or hydromorphone was completely removed) but at least 25 min after delivering the reversal agent. Residual blockade assessment was performed by midlevel providers who were unfamiliar with their interpretation or significance. These providers were nurse practitioners with a master’s or doctorate degree in nursing. Each demonstrated proficiency at the time of accelerometer use instruction, was intermittently reminded of proper use, and could refer to pictures on the accelerometer storage case for proper use.

### 2.6. Extubation Timing

Extubation timing was determined by demonstration of acceptable arterial blood gas values (pH > 7.30, PaCO2 mmHg < 50, PaO2 > 70 mmHg, HCO3 > 17 meq/L) and Rapid Shallow Breathing Index (RSBI) ≤ 80, NIF more negative than −20 cm H_2_O, and FVC 8–10 mL/kg [7] after the patient had been on a pressure support of 5 cm H_2_O, positive end-expiratory pressure of 5 cm H_2_O, and FiO2 0.4 for ≥30 min. The results of the RNB evaluation did not inform the decision to extubate. 

## 3. Results

*Patient Population:* 104 intubated patients (30 Female and 74 male, 63.3 ± 11.7 years old) admitted directly to the ICU following sternotomy and cardiac surgery having not received reversal agents for neuromuscular blockade were admitted to the study (Table 1). Ninety-four patients underwent RNB evaluation both pre-neostigmine administration and pre-extubation. One of these patients had a pre-neostigmine TOFR of 73% and a pre-extubation TOFC 4 recorded, but the post TOFR was not recorded. In an additional 10 patients, providers failed to perform RNB at the post-neostigmine administration time point. No patient required active intraoperative cooling below 33 °C (e.g., circulatory arrest).

**Figure 1:**
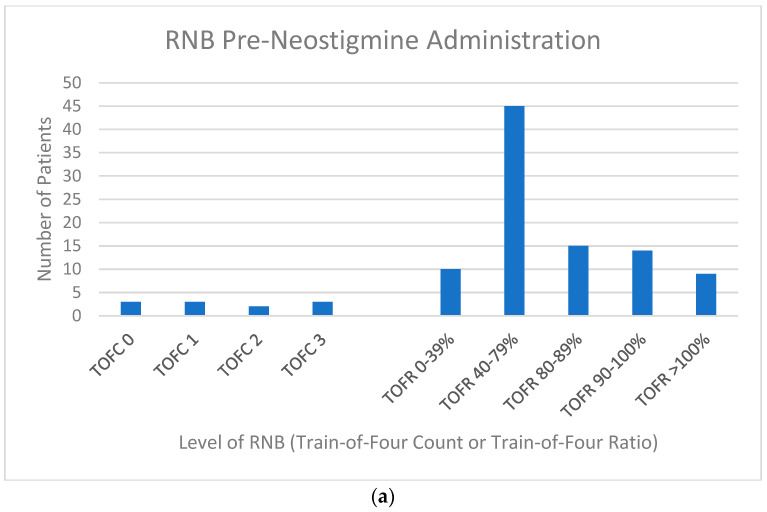

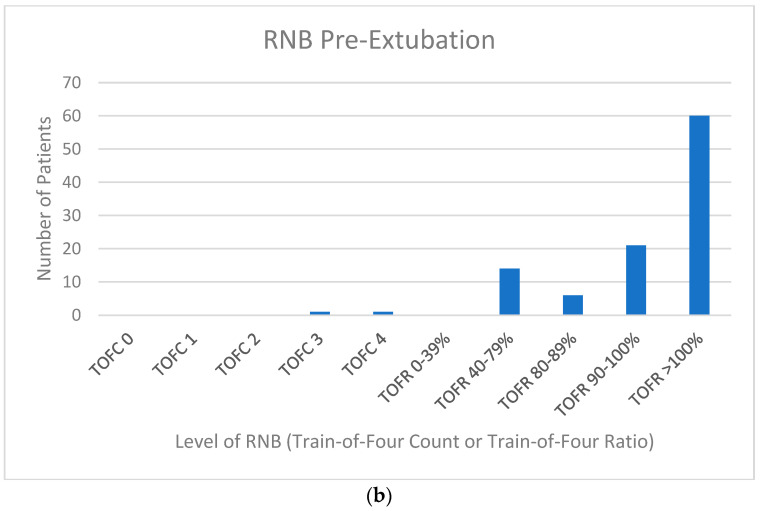
(**a**)—RNB pre-neostigmine administration—histogram indicating number of patients at each Train-of-Four Count (TOFC) or Train-of-Four Ratio (TOFR) as their level of residual neuromuscular blockade (RNB) when analyzed at the preneostigmine administration time point. Total number of patients evaluated = 104. (**b**)—RNB pre-extubation—histogram indicating number of patients at each Train-of-Four Count (TOFC) or Train-of-Four Ratio (TOFR) as their level of residual neuromuscular blockade (RNB) when analyzed at the pre-extubation time point. Total number of patients evaluated = 94.

*Patient Characteristics:* Of the 104 patients, 96 received only rocuronium, 7 received only vecuronium, and 1 received both rocuronium and vecuronium. The timing and quantity of rocuronium dosing and neostigmine dosing, as well as the timing of RNB evaluation, are shown in Table 2. At RNB evaluation prior to neostigmine/glycopyrrolate administration, 93 of 104 patients demonstrated TOFC 4 and generated a TOFR by acceleromyography. Of the remaining 11 patients, 6 demonstrated TOFC < 2 and 5 demonstrate TOFC 2 or 3 (Figure 1a).

**Figure 2:**
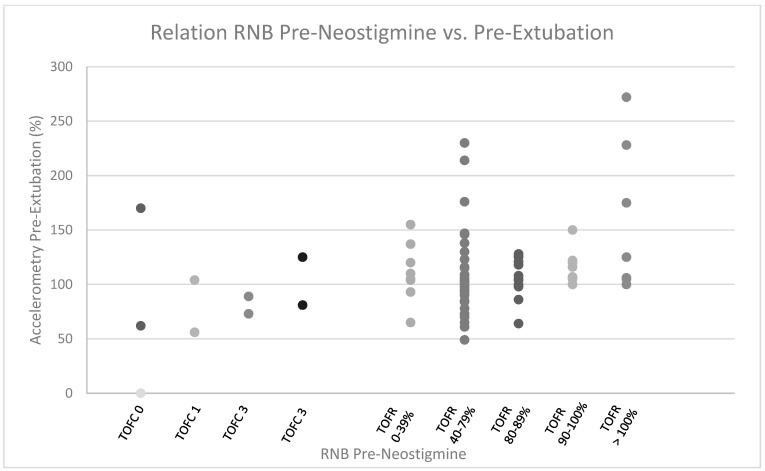
Relation of RNB pre-neostigmine vs. pre-extubation—dot plot indicating the pre-extubation accelerometry level achieved as a function of the level of residual neuromuscular blockade (RNB) present at the time of neostigmine administration. *X*-axis indicates the patients achieving various levels of Train-of-Four Count (TOFC) or Train-of-Four Ratio (TOFR) at the pre-neostigmine administration time point. *Y*-axis indicates the accelerometry level achieved at the pre-extubation time point for individual patients within each pre-neostigmine level of TOFC or TOFR. Accelerometry level of “0” indicates patients whose pre-extubation RNB was less than TOFC 4 and therefore could not be quantified by acceleromyograhphy. Ninety-three of ninety-four patients had RNB values obtained at both pre-neostigmine and pre-extubation time points. One excluded patient had post-extubation TOFC 4 but no indication of TOFR associated with this.

Of the 104 patients, 94 underwent RNB evaluation prior to extubation (Figure 1b). Two of these patients had only qualitative (i.e., TOFC) RNB data recorded—one because the pre-extubation TOFC < 4 prohibited acceleromyography and one with a pre-extubation TOFC 4 but no acceleromyography data recorded. Figure 2 shows the relationship between RNB preneostigmine and RNB pre-extubation in 93 of the 94 patients, with evaluations recorded at both time points. (The patient with a pre-extubation TOFC 4 but no recorded TOFR was excluded.) Of the 93 patients, 10 had pre-neostigmine TOFC < 4 and only qualitative NMB analysis, while 83 had pre-neostigmine TOFC 4 and could therefore have quantitative NMB analysis (i.e., acceleromyography) pre-neostigmine administration. Six of the ten patients (60%) with pre-neostigmine TOFC < 4 failed to achieve TOFR ≥ 90% prior to extubation. Fifteen of the eighty-two patients (18%) with TOFC 4 and acceleromyography on pre-neostigmine evaluation failed to achieve TOFR ≥ 90% prior to extubation. 

The prevalence of patient factors that may compromise post-extubation respiratory mechanics (limited mobility/high inotrope requirement/IABP/OSA) are also listed (Supplemental Information). 

### 3.1. Patients Receiving Unplanned Postextubation Pulmonary Adjunct Support

Three patients received unplanned mechanical ventilatory adjunct support (noninvasive and/or invasive) following extubation. One patient received an unplanned postop regimen of BiPAP alternating with 6l n/c without obvious benefit that began after a planned nighttime BiPAP trial was poorly tolerated. This patient had a BMI of 36.7 and was noncompliant with home BiPAP for known paradoxical breathing. The patient demonstrated a pre-neostigmine TOFR of 68% prior to receiving 0.05 mg/kg neostigmine/0.01 mg/kg glycopyrrolate. Pre-extubation RNB evaluation was TOFR 70%. The patient was extubated 7.17 h following neostigmine administration with NIF −28 cm H_2_O, a vital capacity of 0.63 L, 7.32/45 mmHg/82 mmHg/23 meq/L, and a positive cuff leak but no RSBI recorded on minimal ventilator support.

An arterial blood gas 75 min post-extubation showed 7.33/45 mmHg/90 mmHg/23 meq/L. A planned trial of BiPAP for sleep began 5.5 h following extubation, but patient intolerance and a satisfactory blood gas prior to initiating the trial (7.30/48 mmHg/82 mmHg/23) led to aborting this plan. Unfortunately, the patient’s respiratory acidosis progressed, and the patient began intermittent BiPAP (IPAP 10 cm H_2_O/EPAP 5 cm H_2_O, FiO_2_ 0.4; 2–3 h periods of BiPAP interrupted by 2–3 h periods of 6l n/c) beginning ~12 h following initial intubation. Poor tolerance of BiPAP resulted in a lack of perceived effect (typical ABG off/on BiPAP was similar at ~7.26/52 mmHg/94 mmHg/23 meq/L), in spite of gradually increasing support intensity during the periods it was being applied (IPAP 16 cm H_2_O/EPAP 5 cm H_2_O). All attempts at daytime BiPAP, as well as any blood gas analysis, ended ~60 h following initial extubation. At this point, empiric nighttime BiPAP was utilized without evaluation as to its effectiveness. 

A second patient who received unplanned post-extubation BiPAP had undergone ascending aortic aneurysm repair and left atrial appendage closure, with TEE showing an LVEF of 35–40% on epinephrine 0.05 mcg/kg/min/norepinephrine 0.05 mcg/kg/min following CPB. This patient had a pre-neostigmine TOFR of 61% prior to receiving 0.04 mg/kg neostigmine/0.01 mg/kg glycopyrrolate. No pre-extubation RNB evaluation was performed. The patient was extubated 3.17 h following neostigmine administration with NIF 36 cm H_2_O, RSBI 48 br/min/L), vital capacity of 1.25 L, and 7.35/58 mmHg/134 mmHg/31 meq/L on minimal ventilator support. 

A venous gas 2 h post-extubation showed pH 7.27/70 mmHg/50 mmHg/31 meq/L on 6 l n/c, resp. rate of ~20–22 persistently postop, GCS of 10 persistently postop, and no narcotics other than 50 mcg fentanyl on arrival. At 5 h following extubation, the patient was placed on BiPAP (iPAP 10 cm H_2_O/EPAP 5 cm H_2_O) after arterial 7.24/76 mmHg/160 mmHg/32 meq/L. On BiPAP, ABG recovered to 7.36/54 mmHg/109 mmHg/30 meq/L.

A third patient was reintubated. This patient had a history of bilateral iliac stenting, left carotid-subclavian bypass, and right femoral->axillary bypass. The present surgery was aortic arch repair with aortic debranching (aorta to right carotid, aorta to left carotid, and aorta to left subclavian) and CAB (LIMA->LAD). This patient had a TOFR of 88% prior to administration of 0.05 mg/kg neostigmine/0.01 mg/kg glycopyrrolate. No pre-extubation RNB evaluation was performed. However, the patient demonstrated a NIF of −40 cm H_2_O, a Rapid Shallow Breathing Index of 48 br/min/L, a spontaneous respiratory rate of 18, a vital capacity of 0.73 L, positive cuff leak, and a blood gas of 7.44/41 mmHg/97 mmHg/27 meq/L on PS 5 cm H_2_O, PEEP 5 cm H_2_O, and FiO_2_ 0.4 at 30 min prior to extubation. She was, unfortunately, in the early stages of developing a fever when extubated (temp 38 °C, up 0.4 °C from 15 min earlier) and deteriorated 110 min after extubation to BiPAP support for fever (38.8 °C), respiratory failure (7.24/63 mmHg/72 mmHg/25 meq/L), and eventual intubation 11 h after extubation to return to the operating room for RLE occlusive thrombus.

No patient receiving unplanned supplemental respiratory care had suspected renal or hepatic dysfunction. Two patients had creatinine > 2.0. One, with cr 2.4, had a pre-extubation TOF4 of 110%. One, with cr 3.4, had a pre-extubation TOFr of 155%. One, with cr 2.7, had a pre-neostigmine TOF4 of 97%. None had post-extubation issue.

### 3.2. Patients Receiving Planned Postextubation Pulmonary Adjunct Support

Of the 94 patients having documented RNB evaluations both pre-neostigmine and pre-extubation, 10 received post-extubation adjunct pulmonary care as part of a care plan prepared prior to extubation (Supplemental Information). The reasons included (1) HFNC as part of a protocol to deliver epoprostenol for right ventricular afterload (*n* = 3); (2) HFNC in a patient with arterial pH > 7.35 (*n* = 3); (3) HFNC felt due to hypervolemia (*n* = 2); and (4) extubation on the patient’s home CPAP/BiPAP settings as part of pre-ordained plan (*n* = 2). Only 1 of the 10 demonstrated a pre-extubation RNB < TOFR of 90%. Their pre-extubation RNB was a TOFR of 56%. They underwent planned extubation to epoprostenol via high-flow nasal cannula to reduce afterload on a dysfunctional right ventricle.

## 4. Discussion

Our study was designed to determine whether not using RNB analysis to guide administration of chemical neuromuscular blockade reversal in a postsurgical ICU resulted in consequences related to residual weakness. Of the 104 patients evaluated, 3 patients received unplanned adjunct respiratory care. In at least one of these cases, this unplanned respiratory care (BiPAP) likely reversed progressive respiratory decline related to weakness and possibly prevented reintubation. 

Using observed spontaneous patient movement as the trigger to administer acetylcholinesterase inhibitor to reverse chemical neuromuscular blockade, we failed to recognize that ~12% of patients demonstrated a TOFC < 4, and an additional ~11% of patients demonstrated a TOFR < 40% prior to neostigmine administration. A TOFR of 40% is the minimum level of spontaneous recovery acceptable for antagonism with neostigmine [8,9,10,11]. Furthermore, subjective evaluation failed to recognize that 6% of patients had not yet spontaneously recovered to a TOFC ≥ 2 and that 3% had not yet even recovered to a TOFC ≥ 1. The 2 mg/kg sugammadex dose is Food and Drug Administration (FDA)-approved to antagonize residual rocuronium or vecuronium blockade of TOFC ≥ 2. For TOFC 1 or TOFC 0, sugammadex dosing of 4 mg/kg or 16 mg/kg is FDA-recommended. Neglecting to quantify RNB prior to administering reversal therefore risks not only the inappropriate use of neostigmine but also the inappropriate dosing of sugammadex.

The observed 11% TOFC < 4 at the time of pre-neostigmine dosing was surprising in that the time from the most recent neuromuscular blockade dosing was long (5.1, IQR 3.1–6.4 h), and the rocuronium dosing was not excessive (1.6, IQR 0.6–1.9 mg/kg). Though renal and hepatic functions are critical to the metabolism of rocuronium and vecuronium, renal failure was uncommon, and hepatic failure was nonexistent in our patients preoperatively. Hypothermia can decrease neostigmine efficacy [12], increase rocuronium effectiveness [13], and prolong the metabolism of nondepolarizing neuromuscular blocking agents [14,15]. Though our patients were warmed to ≥36 °C before neostigmine administration, most were <34 °C for at least 35% of their intraoperative course. Improper accelerometer utilization is possible, though the providers utilizing them had been individually trained and demonstrated proficiency before unsupervised use. 

Although 23% of our patients demonstrated RNB above the level recommended before extubation (i.e., TOFR 90%), only 1 of our patients received unplanned pulmonary adjunct care for what was likely RNB. This could, in part, be due to ICU protocols that may help identify those whose RNB is truly dangerous, delaying extubation until RNB is resolved, or even treating patients for RNB. These protocols include evaluating spontaneous breathing patterns and verifying adequate gas exchange with minimal mechanical ventilator support prior to extubation [16], patient positioning, and aggressive pulmonary toilet following extubation, as well as deliberate down-titration of supplemental oxygen support. Compared with many intraoperative scenarios, ICU providers may feel less compelled to urgently extubate patients. This can result in more time being taken to assure RNB resolution prior to extubating patients whose capacity for pulmonary toileting may be limited by positioning restrictions or for whom reintubation is anticipated to be challenging. In our group, 8 patients arrived in the ICU with IABP, and 6 additional patients had known or suspected difficult airways. Furthermore, adjunct respiratory support for purposes other than recognized RNB may simultaneously avoid weakness-related decompensation. One of the ten patients receiving planned adjunct respiratory care in our study demonstrated a pre-extubation TOFR of 56%. The epoprostenol they received via high-flow nasal cannula as part of a planned intervention to reduce right heart afterload may simultaneously have protected against hypoxemia from unrecognized RNB. Finally, ICU-level surveillance identified the need for adjunct mechanical support in at least three patients with unanticipated progressive post-extubation respiratory acidosis. 

Some use post-extubation hypoxemia as a marker of residual neuromuscular blockade [17,18]. Given the multiple potential causes of post-extubation residual A-a gradient in the early post-CPB period, such as hypervolemia [19], systemic inflammatory response [20], and infection [21], hypoxemia in patients capable of standing was considered unrelated to neuromuscular weakness. Nevertheless, the expectation of post-extubation A-a gradient may actually increase the importance of achieving full reversal of chemical neuromuscular blockades. Neuromuscular blocking agents blunt the hypoxic drive in animals [22] and in humans [23]. This attenuated drive is not immediately normalized with complete reversal of chemical neuromuscular blocking agents achieved with either neostigmine or sugammadex [24]. It is tempting to speculate, however, that an earlier return baseline neuromuscular junction activity would hasten normalization of this chemoreflex. 

Quantification of RNB is neither fool-proof nor cost-free. We chose an acceleromyograph with objective quantification of twitch ratio because it is more sensitive than subjective evaluation in detecting postoperative RNB [3,25]. However, RNB evaluation requires repetitive evaluation [26,27], assurance of adequate preload [25], and utilization in a sleeping patient [26] for optimized sensitivity and specificity. Of the 93 patients having pre-extubation NRB evaluation that included documented acceleromyography when appropriate, 7 had pre-extubation RNB evaluation performed within 10 min of extubation (i.e., patient likely awake) (data not shown). Even with attention to these performance measures, acceleromyography may overestimate neuromuscular recovery [28,29]. Furthermore, subjective distress can precede objective decline [30], and subtle risks to airway integrity may be an inherent risk to administration of neuromuscular blocking agents in that they persist even if extubated at a TOFR of ≥90% [31]. Additionally, a simple TOF nerve stimulator costs ~USD 350, while the acceleromyograph used in our study costs ~USD 2450. 

Our study was small and did not address the newly appreciated potential for long-term consequences of residual neuromuscular blockades in susceptible patients such as the elderly [32]. Importantly, our study was carried out by the bedside providers rather than a separate study team. Providers were unaware of the significance of RNB evaluation. Providers found it cumbersome to locate equipment and perform pre-extubation RNB evaluation at a time close enough to the time of extubation to represent recovery, yet while the patient was still receiving adequate sedation and assuring free movement of the hand and fingers. Easily available RNB evaluation equipment, utilizing equipment with repeated reminders of appropriate technique, and provider understanding of its importance will facilitate provider adoption of RNB evaluation, albeit balanced by equipment cost. 

## 5. Conclusions 

In 104 post-cardiac-surgery ICU patients, RNB was reversed with neostigmine/glycopyrrolate administered after patients achieved T > 35.5 °C and were observed making spontaneous respiratory and/or extremity movement efforts. Residual neuromuscular weakness may have been unrecognized before extubation in at least 1 patient, but ICU-level care likely mitigated the consequences. 

## Figures and Tables

**Table 1:** Surgical Procedures: *CAB* Coronary Artery Bypass, *Valve* aortic valve, mitral valve and/or tricuspid valve procedure, *AoV* Aortic Valve, *MV* Mitral Valve, *TV* Tricuspid Valve, *AscAo*/*AoRoot* Ascending Aorta and/or Aortic Root, and *Arch* Aortic Arch.

CAB	CAB/Valve	AoV	MV	TV	AscAo/AoRoot	Arch	Other
58	3	13	13	3	6	2	6 *

* MV/TV × 1, MV/AoV × 1, ASD × 1, LVAD × 2, Ht Transplant × 1.

**Table 2:** Dosing Rocuronium and Timing of RNB Evaluation, *IQR* Interquartile Range, *NMBA* Neuromuscular Blocking Agent, *RNB* residual neuromuscular blockade.

	Rocuronium (mg/kg)	Final NMBA Dose-1st TOF Interval (h)	Neostimine (mg/kg)	Final RNB Evaluation—Extubation Interval (h)
	1.6	5.1	0.045	1.5
IQR	0.6–1.9	3.25–6.75	0.037–0.051	0.12–1.5

## Data Availability

The datasets are available from the corresponding author on reasonable request.

## Supplementary Materials

The following supporting information can be downloaded at: https://www.mdpi.com/article/10.3390/jcm12093253/s1, Supplemental Information.

## Abbreviations

ICU	Intensive Care Unit
TOFC	Train-of-Four Count
TOFR	Train-of-Four Ratio
RV	Right Ventricle
HFNC	High-Flow Nasal Canula
CPAP	Continuous Positive Airway Pressure
BiPAP	Bilevel Positive Airway Pressure
OSA	Obstructive Sleep Apnea
CAB	Coronary Artery Bypass
MV	Mitral Valve
TV	Tricuspid Valve
LVAD	Left Ventricular Assist Device
ASD	Atrial Septal Defect
AscAo	Ascending Aorta
AoRoot	Aortic Root
IABP	Intra-aortic Balloon Pump
OR	Operating Room
RSBI	Rapid Shallow Breathing Index
BMI	Body Mass Index
SD	Standard Deviation
