# Supplementary material for: Reversing Neuromuscular Blockade without Nerve Stimulator Guidance in a Postsurgical ICU—An Observational Study
Source: J Clin Med. 2023 May 2;12(9):3253. doi: 10.3390/jcm12093253 (PMC10179105; doi:10.3390/jcm12093253)
Supplement: Supplementary file 1 [file jcm-12-03253-s001.zip › jcm-2211342-supplementary.pdf]

Supplemental Information

No patient receiving unplanned supplemental respiratory care had suspected renal or hepatic dysfunction. Two patients had creatinine > 2.0. One, with cr 2.4, had pre-extubation TOF4 110%, One, with cr 3.4, pre-extubation TOFr 155%, One, with cr 2.7, pre-neostigmine TOF4 97%. None with post-extubation issue.
We identified 9 patients with possible hepatic compromise. All had TOFr recorded pre-extubation, and no patient had post-extubation respiratory issues felt to result from excess muscle weakness. Distributed by etiology of hepatic compromise, pre-extubation TOFr for 2 patients with ETOH abuse were 180%, 100%*, one patient with illicit drug use was 86%, one patient with viral hepatitis C was 62%, one patient with non-alcoholic fatty liver was 95%, one patient on chronic HIV therapy was 121%, and two patients with possible systemic embolic disease from endocarditis were 92% , 155%.
